# Local Infiltration Analgesia

**Published:** 1899-02-11

**Authors:** 


### Hospital Clinics and Medical Progress.

### LOCAL INFILTRATION ANALGESIA.

For some months past Mr. Arthur Barker[1] has been occasionally operating without using either ether or chloroform, trusting to one or other form of what is now called local infiltration analgesia. A study of the effects produced by the endermic injection of cocaine or eucaine B shows that the primal fact which we have to keep in mind is that cocaine and its salts, applied to the trunk of a sensory nerve, suspend its functions and abolish the sense of pain in all parts supplied by its terminal ramifications. The first who appears to have made practical use of this fact was the French surgeon, Reclus, whose method of producing analgesia by the injection of 1 per cent. cocaine solutions into the soft parts layer by layer does not differ in principle from the methods since developed. But, even with such dilute solutions, toxic effects followed in a few cases, and the application of this method had to be limited to operations confined to a small area. Schleich, however, by further reducing the strength of the solution and by using much greater quantities, was able to extend the method to very much more wide-reaching operations, and now it is employed very largely. Numerous modifications of the method have been employed. One of these, which is only applicable so far to the fingers and the toes, or, perhaps, to the whole hand, consists in arresting the return of blood by applying an elastic ligature round the root of the member in question, and then injecting a very moderate amount of the cocaine solution over each of the several nerves of the part. This arrest of the circulation appears to intensify the action of the drug enormously. Finally, there is the plan of using eucaine B, a drug which is equal to cocaine in analgesic properties, but far less poisonous, dissolved in a saline solution of about the same specific gravity as the blood. Such a solution, consisting of eucaine 1 part, sodium chloride 8 parts, and water 1,000 parts, by weight, is the one now employed by Mr. Barker. It can be used practically in any amount without danger of poisonous effects. As a fact, 300 cc., or 10½ oz., have been injected in an operation without any ill-effects. It is to be observed that the injection of any fluid into the tissues produces certain disturbances. If the temperature is much either above or below that of the body, pain is produced; while if the solutions are of such a nature as to produce osmosis, either from or into the tissues, pain is caused for the moment and then a transient anæsthesia. But if the fluid be indifferent to the tissues as to osmotic power and as to temperature, no pain is produced and no anæsthesia. Such a solution is the one above mentioned, and its analgesic powers depend on its specific effect upon the twigs of the sensory nerves in the tissues through which it is infiltrated. Among other advantages of eucaine B over cocaine are that it is not destroyed by boiling, and therefore can be sterilised, that it keeps well, and is cheap.

The solution is prepared by boiling in seven ounces of distilled water three grains of eucaine B and 24 grains of chloride of sodium. The syringe employed holds 10 c.c., and has an asbestos piston in a glass cylinder. It can be taken to pieces and boiled. The syringe is filled with the above solution at a little above the body temperature, and is armed with the finest needle. "The latter is now thrust obliquely under the epidermis until its eye is buried. A few drops of the solution are then forced out by the screw, when a white plaque is formed. Through this, which is now quite anæsthetic, the needle is forced deeper in various directions while the fluid is pumped out, causing a thorough infiltration of the areola tissue, and, if necessary, of the muscles and periosteum all over the area to be operated on until an artificial œdema is produced, and, presumably, all the nerves of the part are steeped in the fluid. The coarser needles are more suitable for the deeper structures. Finally, the smallest needle is thrust horizontally under the epidermis all along the course of the proposed incision (the screw being turned meanwhile) until a long plaque is produced of white colour and raised." Analgesia is produced in five to ten minutes, and if the operation should be a deep one more fluid must be forced into the deeper tissues in its course. Unless there is some special reason against so doing, as, for example, in cases of obstruction of the bowels, a nutritious meal should be given shortly before operation. Among the disadvantages of the process are that there is no motor paralysis, so that movement may cause embarrassment, that the artificial œdema masks the anatomical details considerably, and the patient remains conscious, which latter fact must greatly limit its use for children and nervous people. For small operations it certainly has a large field of utility. It does not produce total anæsthesia or motor paralysis, but only analgesia.

[1] Lancet, Feb. 4.

